# Outcomes of Bone Marrow Mononuclear Cell Transplantation for Neurological Sequelae Due to Intracranial Hemorrhage Incidence in the Neonatal Period: Report of Four Cases

**DOI:** 10.3389/fped.2019.00543

**Published:** 2020-01-24

**Authors:** Nguyen Thanh Liem, Truong Linh Huyen, Le Thu Huong, Ngo Van Doan, Bui Viet Anh, Nguyen Thi Phuong Anh, Dang Thanh Tung

**Affiliations:** ^1^Cellular Manufacturing Department, Vinmec Research Institute of Stem Cell and Gene Technology, Hanoi, Vietnam; ^2^Vinmec Times City General Hospital, Hanoi, Vietnam

**Keywords:** intracranial hemorrhage, neurological sequelae, bone marrow mononuclear cells, autologous, neonate

## Abstract

**Aim:** The aim of this study was to present primary outcomes of autologous bone marrow mononuclear cell (BMMNC) transplantation to improve neurological sequelae in four children with intracranial hemorrhage (ICH) incidence during the neonatal period.

**Methods:** GMFM88 and modified Ashworth score were used to assess motor function and muscle spasticity before BMMNC transplantation and after transplantation. Brain MRI was performed to evaluate brain morphology before and after BMMNC transplantation. Bone marrow were harvested from anterior iliac crest puncture and BMMNCs were isolated using Ficoll gradient centrifugation. The microbiological testing, cell counting, and hematopoietic stem cell (hHSC CD34+ cell) analysis were performed, following which BMMNCs were infused intrathecally.

**Results:** Improvement in motor function was observed in all patients after transplantation. In addition, muscle spasticity was reduced in all four patients.

**Conclusion:** Autologous BMMNC transplantation may improve motor function and reduce muscle spasticity in children with ICH incidence during the neonatal period.

## Introduction

Intracranial hemorrhage (ICH), including extra-ventricular hemorrhage and intra-ventricular hemorrhage, is a severe condition in neonates, especially in preterm babies with high morbidity and mortality. It severely affects the central nervous system because the neonatal period is a critical window for brain development (1, 2).

Intra-ventricular hemorrhage is the common type of ICH in premature newborns. The incidence of intra-ventricular hemorrhage among infants born between 24 and 31 complete weeks in China in 2013–2014 was 15.4% (3), whereas the rate of severe intra-ventricular hemorrhage among infants born between 22 and 31 weeks of gestation at hospitals in California, United States, between 2005 and 2015 was 7.7% (4). Different mechanisms are cited to explain the etiology of intra-ventricular hemorrhage in premature babies, including fragility of the germinal matrix vasculature, disturbance in the cerebral blood flow, and coagulation disorders. Many factors such as a low gestational age and birth weight, intrauterine infection, vaginal delivery, low Apgar score, severe respiratory distress syndrome, pneumothorax, hypoxia, hypercapnia, seizures, patent ductus arteriosus, and thrombocytopenia are thought to be risk factors associated with intra-ventricular hemorrhage (5–7).

ICH occurred not only in preterm infants but also in full-term babies related to brain trauma, vascular malformation, vitamin K deficiency, and many other conditions (1, 8–13).

ICH in neonates results in severe neurodevelopmental adverse effects. Bolisetty et al. showed that infants born at 23 to 28 weeks' gestation with grade III–IV intra-ventricular hemorrhage had high rates of developmental delay (17.5%), cerebral palsy (30%), deafness (8.6%), and blindness (2.2%) (2). According to Pierrat et al., the overall rate of cerebral palsy in infants born at 24–26, 27–31, and 32–34 weeks of gestation was 6.9, 4.3, and 1%, respectively (14).

Traditional treatments of neurological sequelae after ICH in newborns have limited effect. Recently, stem cell transplantation is being applied in the management of neurological impairment in children, resulting in promising outcomes (15–21). In 2018, Ahn et al. published the first report using stem cell transplantation for severe intra-ventricular hemorrhage to reduce neurological complications (22). However, to our knowledge there have been no publications using stem cell transplantation in the management of neurological sequelae after ICH incidence in the neonatal period.

The aim of this report is to present outcomes of bone marrow mononuclear cell (BMMNC) transplantation in four children with neurological sequelae due to ICH incidence during the neonatal period.

## Patients and Methods

### Patient's Enrollment

The study was approved by the Hospital ethical committee and performed at Vinmec International Hospital in Hanoi, Vietnam. The parents were carefully educated about anticipated benefits and potential side effects of the procedures and provided written informed consent according to the Good Clinical Practice and the Helsinki Declaration. The general data collected before the transplantation consisted of age, gender, past medical history, clinical findings on admission, and brain MRI manifestations.

Prior to the treatment, the patients were thoroughly examined by an experienced physiotherapist using Gross Motor Function Classification System (GMFCS), Gross Motor Function Measure-88 (GMFM-88) scores, and modified Ashworth scale, and by an experienced psychologist using Denver II scale (23–25).

Four children with neurological sequelae due to ICH incidence in the neonatal period were enrolled in this study. Written informed consent was obtained from the minor(s)' legal guardian/next of kin for the publication of any potentially identifiable images or data included in this article.

### BMMNC Procedure

#### Bone Marrow Aspiration

Bone marrow was harvested through anterior iliac crest puncture under general anesthesia in the operating theater. The volume collected depended on the patients' body weight as follows: 8 ml/kg for patients under 10 kg [80 ml + (body weight in kg – 10) × 7 ml] for patients above 10 kg (18).

#### BMMNC Isolation, Characterization, and Preparation for Transplantation

BMMNCs were isolated by gradient centrifugation using Ficoll-Paque (GE Healthcare, Sweden) in a clean room following ISO 14644 standard at Vinmec Research Institute of Stem Cell and Gene Technology. The cell suspension was washed with phosphate-buffered saline (PBS) solution and re-suspended in autologous plasma up to a total of 10 ml for injection. The sterility of the product was confirmed by microbiological evaluation by BacT/Alert® 3D microbial detection System (Biomerieux, USA). The total blood components before and after the Ficoll-Paque separation was evaluated by Beckman Coulter LH780 hematocytometer. The hematopoietic stem cell content (hHSC CD34+) was assessed according to the International Society of Hematotherapy and Graft Engineering guideline (ISHAGE guideline) using Stem-Kit™ Reagent, Beckman Coulter in Navios flow cytometer. Before injection, cell products were examined for endotoxin levels by using the Endosafe-PTS kit (Charles River).

#### BMMNC Infusion

BMMNCs were infused intrathecally between the 4th and 5th lumbar vertebrae over the course of 30 min using an electrical pump, through an 18-gauge spinal needle.

### Follow-Up Assessment

The GMFM-88 was applied to evaluate improvement of motor function, modified Ashworth scale was used to assess muscle spasticity, and child development was measured by Denver II testing (24–26).

### Case Presentation

#### Case 1

A 2,900-g boy was born at 40 weeks' gestation by C-section on 29th October 2014 due to prolonged labor. Asphyxia occurred right after birth, and ICH was detected at 1 day postpartum. Respirator support with a ventilator plus medical treatment was required for 3 weeks. Motor skill milestones were delayed and cerebral palsy was diagnosed at 5 months of age. Since then, the patient underwent 2 h of physiotherapy per day.

**Figure 1:**
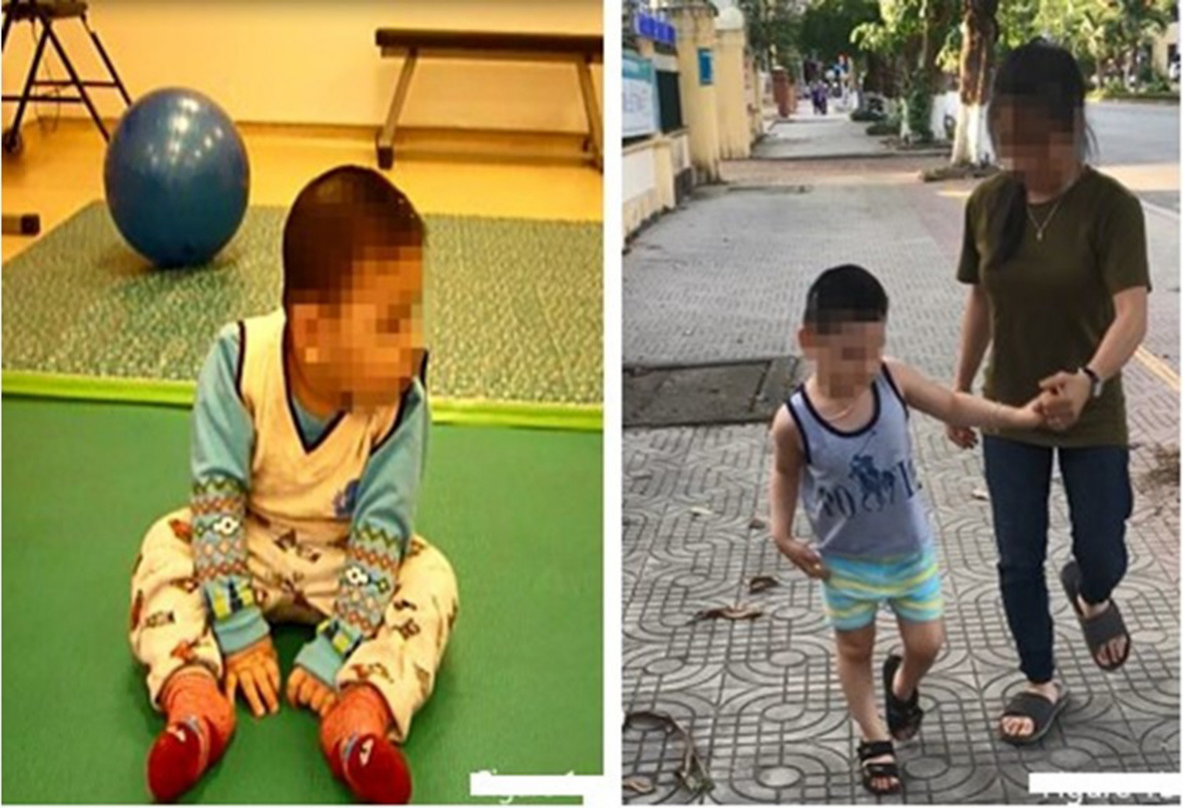
The improvement of motor functions and muscle tone in Case 1 before and after stem cell transplantation.

Examination on 10th December, 2015 when he was 14 months old, confirmed cerebral palsy at severe level (GMFCS level V) with mental retardation, tetraplegia, poor motor function skills, muscle spasticity, retracted Achilles tendons, and dysphagia. He could sit down with arm propping but could not crawl or stand (Figure 1—*A permission from his mother was obtained to let his image be published*). His vision was impaired, in which he could only distinguish between dark and light at very short distance. Brain MRI showed diffuse cortical and sub-cortical lesions on both sides (temporal lobe, parietal lobe, occipital lobe, and one part of frontal lobe) with diffuse brain atrophy, dilation of third ventricle, and bilateral ventricles.

The patient underwent four BMMNC transplantations at the age of 14, 17, 24, and 39 months with cellular information as presented in Table 1. No adverse effects were observed. After discharge, physiotherapy was given 2 h per day for 12 days at the rehabilitation department and then continued at home.

**Table 1:** Details in BMNMCs transplantations.

**Patient**	**Transplantation**	**Date**	**Age (months)**	**Total** **MNCs**	**Total** hHSC **(CD34+)**
1	1st	11/12/2015	14	147 × 10^6^	18.55 × 10^6^
	2nd	11/03/2016	17	105 × 10^6^	13.25 × 10^6^
	3rd	26/10/2016	24	553 × 10^6^	67.40 × 10^6^
	4th	18/01/2018	39	630 × 10^6^	57.75 × 10^6^
2	1st	02/08/2016	47	588 × 10^6^	48.05 × 10^6^
	2nd	14/02/2017	53	866 × 10^6^	40.30 × 10^6^
	3rd	28/08/2017	59	672 × 10^6^	35.49 × 10^6^
3	1st	06/12/2017	15	300 × 10^6^	37.20 × 10^6^
4	1st	19/05/2017	68	590 × 10^6^	40.55 × 10^6^
	2nd	27/11/2017	74	490 × 10^6^	19.95 × 10^6^

*BMMNCs, Bone marrow mononuclear cells; MNCs, Mononuclear cells; hHSC, human Hematopoietic Stem Cell*.

#### Progress After Transplantations

Improvements of motor functions and muscle tonus were observed after transplantations (Table 2). The patient was observed to be capable of sitting and crawling at 3 months after the first transplantation, standing by holding furniture at 10 months after the first transplantation, and walking with support by holding parents' hand since 25 months after first transplantation (Figure 1). Muscle spasticity was reduced from 2 to 1 on the modified Ashworth scale. Dysphagia disappeared since 10 months after the first transplantation.

**Table 2:** Improvement of motor function, muscle spasticity, and Denver II after transplantation.

	**Patient 1**		**Patient 2**		**Patient 3**		**Patient 4**	
	**Before**	**After**	**Before**	**After**	**Before**	**After**	**Before**	**After**
Modified Ashworth	2	1	4	2	2	1	4	2
GMFCS	5	2	5	3	2	1	4	3
**GMFM-88**								
Lying, rolling	28	51	24	51	49	51	40	47
Sitting	18	60	7	49	53	60	42	58
Crawling and Kneeling	0	39	0	8	33	42	10	37
Standing	0	12	0	10 (12 with KAFO)	2	39	6	12
Walking, running and jumping	0	18	0	8 (23 with KAFO)	0	60	6	12
**Denver II (months)**								
Personal—social	3	12	7	8	11	22	24	30
Fine motor—adaptive	3.5	9	–	–	–	–	–	–
Right hand	–	–	3	4	5	12	6	6
Left hand	–	–	6	7	11	23	30	42
Reception language	5	15	8	12	14	23	50	52
Expressive language	3	9	5	6	14	23	50	52
Gross motor	5	12	7	10	10	24	12	13

His vision was well improved from recognizing the light from mobile phone or other tools at 10 months after transplantation to identifying and finding objects of his desire at 25 months after first transplantation.

Brain MRI taken on 15th January 2018, when he was 39 months old, showed that dilatation of ventricles was reduced in comparison with pre-transplantation brain MRI.

#### Case 2

A boy was born on 10th September 2012 at 32 weeks' gestation by emergency C-section due to Oligohydramnios and Chorioamnionitis with a birth weight of 1,800 g. He suffered from perinatal asphyxia and ICH was diagnosed at 1 day after birth. He required respiration support with ventilator for 10 days and then oxy mask for 2 weeks. Ventriculoperitoneal shunt was inserted at 2 months of age due to hydrocephalus. His motor skills were delayed and cerebral palsy were confirmed at 9 months of age. Since then, he underwent 3 h of physiotherapy per day.

Examination on 1st August 2016, at 47 months old, showed cerebral palsy at severe level (GMFCS level V) with mental retardation, tetraplegia, poor gross motor function skills and poor fine motor skill, poor head control, muscle spasticity, stiffness bent arms, wrist deformities, retracted Achilles tendons, and dysphagia. He could roll, but could not sit up.

Seizures occurred 1–2 times per week requiring antiepilepsy medicament (Depakine) with a dose of 200 mg two times per day.

**Figure 2:**
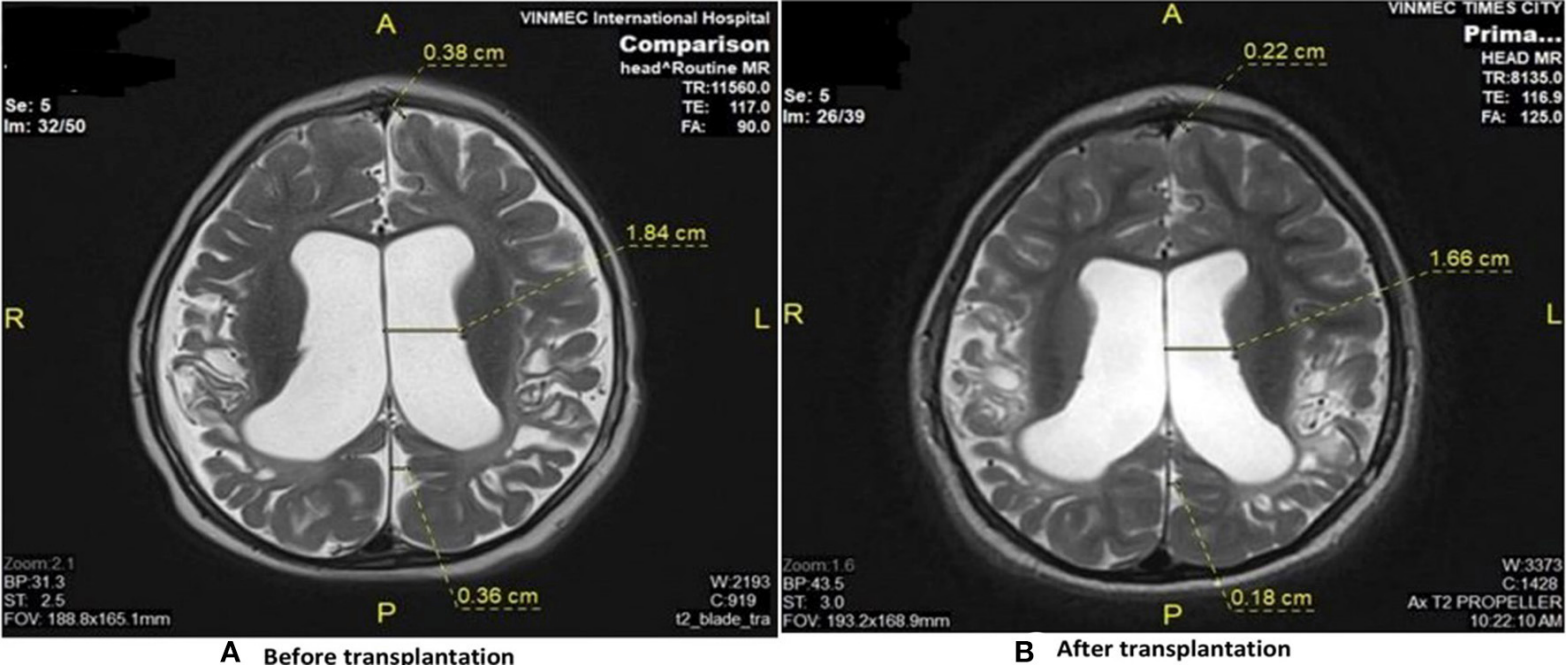
Brain MRI results revealed the improvement before and after stem cell transplantation. **(A)** Diffuse cortical and subcortical lesions with diffuse brain atrophy and dilated ventricles before transplantation. **(B)** Diffuse brain atrophy, and dilation of ventricles reduced after transplantation.

Brain MRI demonstrated diffuse cortical and sub-cortical lesions on the left sides (temporal lobe, parietal lobe, occipital lobe and frontal lobe) with diffuse brain atrophy dilated bilateral ventricles (Figure 2).

The patient underwent three BMMNC transplantations at the age of 47, 53, and 59 months without any adverse effects. The details of each cell transplantation are shown in Table 1. After discharge, physiotherapy was given 2 h per day at the rehabilitation department for 12 days and then continued at home.

#### Progress After Transplantations

Improvement of the gross motor function and fine motor skills were observed at 6 months after the first transplantation. He could sit up with support on the wall or stand with holding furniture.

These motor functions continuously improved significantly, as he could stand without support, walk with support by parents holding hands or wheels pulpit frame at 22 months after the first transplantation. The improvement in motor function measured by GMFM-88 before and after three transplantations is shown in Table 2.

Muscle spasticity was reduced. At 22 months after the first transplantation, the modified Ashworth score reduced from level 4 to 2 (Table 2). Stiffness bent arms, wrist deformities, and retracted Achilles tendons were improved.

Dysphagia subsided at 6 months after the first transplantation and disappeared thereafter so he could eat solid food.

Depakine was reduced from 200 mg per day to 150 mg per day since 12 months after the first transplantation and then was stopped since 22 months without seizure.

Brain MRI taken on 11th June 2018 when he was 69 months old showed diffuse brain atrophy of supratentorial region and slightly decreased dilated third ventricle and bilateral ventricle and subarachnoid space compared to MRI study date 2nd June 2016 (Figure 2).

#### Case 3

A 3,300-g boy was born on 27th September 2016, at 42 weeks' gestation by emergency C-section because of prolonged labor. At 33 days old, he suddenly suffered from cyanosis and then coma. ICH was diagnosed and an operation was performed to remove a hematoma. Since 6 months old, hydrotherapy was applied 3 days per week, 30 min per day.

Examination on 4th December 2017 (15 months old) showed cerebral palsy at level II of GMFCS with right side hemiplegia, poor fine motor function at right hand, muscle spasticity, right stiffness bent arm, hand and wrist deformities, and jumping knee gait. He could stand with holding furniture but could not step.

Brain MRI revealed diffuse cortical and sub-cortical lesions on the left sides (temporal lobe, parietal lobe, occipital lobe, and frontal lobe). Periventricular leukomalacia adjacent to the right lateral ventricular, dilatation of the left lateral ventricular, diffuse corpus callosum atrophy.

The patient underwent a BMMNC transplantation at the age of 15 months with the cell dose of 300 × 10^6^ MNCs and 37.2 × 10^6^ hHSCs (CD34+). No adverse effects were observed. He continued to receive the same regimen of physiotherapy.

#### Progress After Transplantation

Significant improvement of motor functions and muscle spasticity were observed at 7 months after the first transplantation. The patient could walk, jump, and climb stairs without support. Right stiffness bent arm was improved; no more hand and wrist deformities or jumping knee gait. The improvement in motor function and Denver II score was presented in Table 2. There were no significant changes on brain MRI taken on 26th July 2018 compared to brain MRI before the transplantation.

#### Case 4

A boy was born on 16th September 2011, at 40 weeks' gestation by emergency C-section due to placenta previa with a birth weight of 2,900 g. At 28 days after birth, he suffered from severe vomiting, cyanosis, and then coma, requiring respiration support with ventilator. ICH was diagnosed and managed medically without operation for 20 days.

Examination on 18th May 2017 (68 months old) confirmed cerebral palsy at level IV of GMFCS with mental retardation, tetraplegia, poor fine motor function, muscle spasticity, stiff knee gait, jumping knee gait, retracted Achilles tendons, stiffness bent arms, and hand and wrist deformities. He could sit without support but could not crawl, and could not stand either.

Brain MRI revealed diffuse cortical and sub-cortical lesions on the left sides (temporal lobe, parietal lobe, occipital lobe, and frontal lobe) and the right frontal lobe, dilated left lateral ventricular, and diffuse corpus callosum atrophy.

The patient underwent two BMMNC transplantations at the age of 68 and 74 months, respectively, without any adverse effects, and received the same physiotherapy regimen. The details for each cell transplantation are presented in Table 1.

Motor function in four patients with intracranial hemorrhage in the neonatal period before and after bone marrow mononuclear cell transplantation was displayed in Supplementary Videos 1–4, respectively.

#### Progress After Transplantations

His gross motor function improved, enabling him to take steps by holding onto furniture and walking with support by parents holding hand at 6 months after first transplantation. At 14 months after the first transplantation, he could stand without support, and walked with hand holding. Retracted Achilles tendons, stiffness bent arms, stiffness knee gait, and jumping knee gait were improved at 14 months after the first transplantation. The muscle spasticity was reduced from 4 to 2 on the modified Ashworth scale. The improvement in motor function and Denver II score is shown in Table 2.

Brain MRI taken 14 months after the first transplantation when he was 82 months old did not show any significant changes compared to pre-transplantation brain MRI.

## Discussion

ICH causes immediate and delayed injury to the brain. Mass effect of hematoma, increased intracranial pressure, decreased cerebral perfusion pressure, released inflammatory cytokines, and free radicals result in ischemia and hypoxia of the brain parenchyma, white matter injury, and periventricular leukomalacia (27). These effects cause many severe neurological sequelae such as cerebral palsy, seizures, cognitive deficits, hydrocephalus, and death (2, 14).

Physical therapy is a standard treatment. However, Harries et al. showed that only 22% of children with severe cerebral palsy could improve after physical therapy (28). The contribution of physical therapy is to reduce the adverse effects of disuse, inexperience, and secondary complications (29).

To improve neurological sequelae after intra-ventricular hemorrhage, many research efforts using stem cells have been carried out in animals (30–32). Ahn et al. performed intra-ventricular transplantation of umbilical cord blood-derived mesenchymal stem cells (MSCs) for induced intra-ventricular hemorrhage in Newborn Sprague–Dawley rats. They noticed that stem cells can significantly attenuate the post hemorrhagic hydrocephalus and brain injury after intra-ventricular hemorrhage (30). Wang et al. carried out an experimental study to assess the effectiveness of bone marrow MSC transplantation for rats with induced intracranial hematoma. The results suggested that intravenous BM-MSC infusion exerts therapeutic effects on ICH in spontaneously hypertensive rats (32).

In rodents with experimental ICH, Suda et al. also found that autologous BMMNC transplantation can improve long-term structural and functional outcomes (31).

Bone marrow contains a population of pluripotent cells that can differentiate into a variety of cell lineages, including neural cells, and MSCs could secrete neurotrophic factors, anti-inflammatory cytokines, and angiogenic factors to assist in nerve repair and regeneration, thereby facilitating neural stem cell growth and differentiation (33). Whole bone marrow-derived mononuclear cells (BMMCs), which are a mixture of undifferentiated cells including both HSCs and MSCs, could potentially produce a better and synergic effect as compared to each cell line alone. Furthermore, autologous BMMCs can be readily isolated from bone marrow and have no risk of rejection and no ethical restrictions.

Based on results of experimental studies, the role of stem cell in management of ICH in humans is being investigated. Sobrino et al. found that the higher level of circulating HSCs at admission and at 7 ± 1 days after ICH was associated with the better functional outcome at 3 months (34). England et al. used G-CSF to mobilize HSCs into the circulation and found that HSCs could promote functional recovery from not only ischemic stroke but also ICH (35).

Zhu et al. compared outcomes of surgery combined with autologous bone marrow stromal cell transplantation vs. surgery alone for treatment of intra-cerebral hemorrhage. The findings of this trial showed that surgery combined with autologous bone marrow stromal cell transplantation is safe, providing short-term therapeutic benefits for ICH (36).

Four patients in our report had ICH during the neonatal period. In two patients, ICH occurred on the first day after birth related to premature birth and asphyxia. In two other patients, ICH happened at later stages (28 and 33 days after birth) that may be related to vitamin K deficiency.

Four patients underwent BMMNCs transplantations without occurrence of any adverse side effects, indicating this treatment to be safe. Improvement of different aspects including muscle spasticity and motor function after BMMNC transplantations were observed in all four patients.

Before BMMNC transplantation, neither patient could walk but they were able to walk after transplantation. GMFCS decreased three levels in patients 1 and 2, and one level in patients 3 and 4. It suggests that BMMNC transplantation affords patients requiring less support as they become more independent due to significant improvement of gross motor function.

The total GMFM-88 score and the scores of all component domains increased after the intervention in all four patients. The improvement was observed not only in motor function but also in the fine motor skill in all patients. Personal–social, fine motor–adaptive, and language skills were improved after transplantations.

All patients had muscle spasticity before BMMNC transplantation (two patients at level 4 and two patients at level 2). However, muscle spasticity decreased remarkably after transplantation in all of them. That reduction could contribute to the improvement of motor functions.

Two patients suffered from dysphagia before transplantation, which ceased after BMMNC transplantation. Dysphagia could be caused by spasticity of muscles that coordinate swallowing activities. Disappearance of dysphagia could be related to reduction of muscle spasticity after BMMNC transplantation.

One patient had associated epilepsy, which ceased after transplantation. This result has not been mentioned in any previous reports. This suggests that stem cell transplantation may have beneficial effects to associated epilepsy in patients with neurological sequelae after ICH.

One patient of our cohort had visual impairment, which was improved after stem cell transplantation. Li et al. also reported one patient who had visual impairment associated with cerebral palsy whose impaired vision improved after stem cell transplantation (37). The question whether stem cell transplantation can repair impaired vision in patients with neurologic sequelae after ICH requires additional research in the future to further verify this outcome.

In this study, important clinical improvements have been observed in all patients. However, significant changes of brain MRI were observed only in one case whose dilatation of ventricles was reduced after the transplantation. It suggests that stem cells may improve brain function but may not result in significant changes in brain morphology. That is why a more sensitive tool like PET CT or PET MRI should be used to assess functional changes of the brain after stem cell transplantation in future studies.

## Conclusion

In conclusion, our results showed that BMMNC transplantation may improve neurological sequelae due to ICH incidence during the neonatal period. However, studies with larger patient numbers should be carried out to achieve a more accurate conclusion.

## Data Availability Statement

All datasets generated for this study are included in the article/Supplementary Material.

## Author Contributions

NL: conception and design, data analysis and interpretation, manuscript writing, and final approval of manuscript. TH: collection and assembly of data, data analysis and interpretation, and manuscript writing. LH: provision of study material or patients, collection, assembly of data, and manuscript writing. ND, BA, NA, and DT: provision of study material or patients, collection, assembly of data, data analysis and interpretation.

### Conflict of Interest

The authors declare that the research was conducted in the absence of any commercial or financial relationships that could be construed as a potential conflict of interest.

## Supplementary Material

Supplementary Video 1 Motor function in case 1 with intracranial hemorrhage in the neonatal period before and after bone marrow mononuclear cell transplantation.

Supplementary Video 2 Motor function in case 2 with intracranial hemorrhage in the neonatal period before and after bone marrow mononuclear cell transplantation.

Supplementary Video 3 Motor function in case 3 with intracranial hemorrhage in the neonatal period before and after bone marrow mononuclear cell transplantation.

Supplementary Video 4 Motor function in case 4 with intracranial hemorrhage in the neonatal period before and after bone marrow mononuclear cell transplantation.
